# Recognizing Risks and Optimizing Perioperative Care to Reduce Respiratory Complications in the Pediatric Patient

**DOI:** 10.3390/jcm9061942

**Published:** 2020-06-22

**Authors:** Chinyere Egbuta, Keira P. Mason

**Affiliations:** Harvard Medical School, Boston Children’s Hospital, Department of Anesthesiology, Critical Care and Pain Medicine, 300 Longwood Ave., Boston, MA 02115, USA; Chinyere.Egbuta@childrens.harvard.edu

**Keywords:** perioperative, complication, pediatric, respiratory, risk factors

## Abstract

There have been significant advancements in the safe delivery of anesthesia as well as improvements in surgical technique; however, the perioperative period can still be high risk for the pediatric patient. Perioperative respiratory complications (PRCs) are some of the most common critical events that can occur in pediatric surgical patients and they can lead to increased length of hospitalization, worsened patient outcomes, and higher hospital and postoperative costs. It is important to determine the various factors that put pediatric patients at increased risk of PRCs. This will allow for more detailed and accurate informed consent, optimized perioperative management strategy, improved allocation of clinical resources, and, hopefully, better patient experience. There are only a few risk prediction models/scoring tools developed for and validated in the pediatric patient population, but they have been useful in helping identify the key factors associated with a high likelihood of developing PRCs. Some of these factors are patient factors, while others are procedure-related factors. Some of these factors may be modified such that the patient’s clinical status is optimized preoperatively to decrease the risk of PRCs occurring perioperatively. Fore knowledge of the factors that are not able to be modified can help guide allocation of perioperative clinical resources such that the negative impact of these non-modifiable factors is buffered. Additional training in pediatric anesthesia or focused expertise in pediatric airway management, vascular access and management of massive hemorrhage should be considered for the perioperative management of the less than 3 age group. Intraoperative ventilation strategy plays a key role in determining respiratory outcomes for both adult and pediatric surgical patients. Key components of lung protective mechanical ventilation strategy such as low tidal volume and moderate PEEP used in the management of acute respiratory distress syndrome (ARDS) in pediatric intensive care units have been adopted in pediatric operating rooms. Adequate post-operative analgesia that balances pain control with appropriate mental status and respiratory drive is important in reducing PRCs.

## 1. Introduction

Despite significant advancements in the safe delivery of anesthesia and improvements in surgical technique, the perioperative period can still be fraught with risks for the pediatric patient. To date, there is no clearly defined definition for perioperative respiratory complication (PRC). For this review, PRC will comprise of any adverse event that affects the respiratory system occurring either in the intraoperative or the postoperative period. PRCs can lead to increased length of hospitalization, worsened patient outcomes, and higher hospital and postoperative costs [1,2,3,4,5,6].

This paper will present a comprehensive review of the most recent literature on PRCs in pediatric patients. The authors searched PubMed, Medline, and the Boston Children’s Hospital medical e-library using the following search terms: postoperative/perioperative/post-surgical respiratory complications solely at first, then further specifying pediatric patients. The authors also used supplementary search methods such as assessing the similarly articles section in PubMed search results and the reference lists of selected studies. The authors included both pediatric and adult studies that are most relevant to clinical practice, while highlighting studies that were published after 2000. Although most of the literature on PRC focus on adult patients, understanding this population’s perioperative risk factors, presentation and outcome can, in many cases, aid in and be applicable to the pediatric population. Understanding the perioperative risk factors for PRCs can provide an invaluable opportunity to create and deploy targeted interventions to reduce perioperative morbidity and mortality [7,8].

## 2. Definition

There is no standardized definition of PRC in the pediatric literature. This variability in definition makes it challenging to understand the true incidence and outcome of PRCs, as each study determines the frequency of the respiratory events based on a non-standardized definition, often created for the purpose of the study. To delineate the events that can define a PRC, the European Society of Anaesthesiology assembled a joint taskforce to standardize definitions and outcome measures for perioperative medicine. These definitions were entitled European Perioperative Clinical Outcome (EPCO) definitions and were intended to be used for clinical effectiveness research. Standardized definitions were presented for Respiratory infection, Respiratory failure, Pleural effusion, Atelectasis, Pneumothorax, Bronchospasm, Aspiration pneumonitis, and Pneumonia [9]. To date, no such initiative has been done that is specifically related to pediatrics. Consequently, the literature of pediatric perioperative outcomes include random definitions for laryngospasm, bronchospasm, apnea/hypopnea, hypoxemia or oxygen requirement, hypercarbia or hypoventilation, aspiration airway obstruction, cough, or reintubation (see Table 1 for the published definitions of PRCs in the current pediatric anesthesia literature post-2000).

## 3. Incidence

PRCs are some of the most common critical events that can occur in pediatric surgical patients [6,9,10,11,12,13,14,15,16,17,18,19]. It has been estimated that of all the perioperative damaging events (297 damaging events per 10,000 anesthetics), over 75% involve the respiratory system, of which 36% are related to laryngospasm [19]. In 2013, the Wake-Up Safe (WUS) initiative concurred: Respiratory etiology for Serious Adverse Events (SAE) represented 34% of the overall 1.4 per 1000 pediatric anaesthetics [13]. Table 1 summarizes the varied incidence of PRCs, which has been reported in the literature (0.1 to 3% of anesthetics) since 2000.

## 4. Cost, Morbidity and Mortality

The Agency for Healthcare Research and Quality (AHRQ) developed Patient Safety Indicators (PSI), reviewing in-hospital data up to time of discharge, in order to identify potential pediatric inpatient safety issues [2]. They reported postoperative respiratory failure in 33 per 1000 pediatric patients (0 to 18 years) [2]. The financial burden of these events were significant: An average of 24.4 additional days in the hospital, $140,507 in increased charges and an increased risk of in-hospital mortality (Odds Ratio 76.6) [2]. A similar study was conducted, examining outcome data after hospitalization at 38 freestanding pediatric, academic, not-for-profit, tertiary care hospitals. In these settings, postoperative respiratory failure was a leading cause of hospital expenses: an average of 4.8 extra days in-hospital and excess total charges of $77,739 on average [1].

Malpractice closed claims offer important insights into the types of events that lead to morbidity and mortality. There is a significantly higher rate of closed claims related to PRCs for pediatrics than adults with substantial morbidity, mortality and successful monetary awards [6]. In a review of 69 New York State insurance claims and 87 national court trials alleging injury related to tonsillectomy, of which there were 38 deaths/major injury with 36 cases presenting an identifiable etiology, PRCs represented 13 out of the 36 identifiable causes of death/major injury [20].

## 5. Preoperative Risk Assessment and Stratification

The anatomy of a child’s airway evolves quite dramatically from birth to adolescence. The proximity of pharyngeal structures combined with smaller mandibles, smaller tracheal diameter as well as relatively large tongues lead to significantly increased risk of airway obstruction in infants and young children. It is important to identify the various factors that predict an increased risk of pediatric PRCs. This identification will improve our ability to advise patients for informed consent, optimize perioperative management strategy, improve allocation of clinical resources, and, identify PRCs for quality metrics and research purposes. Although there are published studies describing risk factors for PRCs, there remains a paucity of validated predictors and models for pediatrics. In the following section, the preoperative and perioperative risk factors which have been examined as posing an increased risk for PRCs will be evaluated.

### 5.1. Pediatric Preoperative Risk Prediction Tools

#### 5.1.1. Snoring, Trouble Breathing, and Un-Refreshed (STBUR) Questionnaire

The STBUR Scale is a 5-item questionnaire that has been developed from the Sleep-Related Breathing Disorder (SRBD) questionnaire, which has been validated in children with sleep disordered breathing (SDB) [21]. A prospective study of 337 parents compared responses on the SRBD questionnaire with the occurrence of Perioperative Respiratory Adverse Events (PRAE) which included major cough, major breath-hold, laryngospasm, bronchospasm, airway obstruction, major desaturation and critical PRAE [21]. The STBUR tool was created by identifying the five symptoms from the SRBD questionnaire which were strongly predictive of the occurrence of PRAE. The positive likelihood ratio of PRAE was 3.06 (three-fold higher) in the presence of any 3 STBUR symptoms, 9.74 (ten-fold higher) with 5 STBUR symptoms, and 2.6 when using only polysomnography (PSG)-confirmed diagnosis of SDB [21].

The STBUR Questionnaire could replace the SRBD and PSG by offering a more accurate method to predict children at risk for SDB and PRAE [6,21].

#### 5.1.2. Perioperative Respiratory Adverse Events in Pediatric Ambulatory Anesthesia: Risk Prediction Tool

A large study of 19,059 children (age < 18 years) were divided into two cohorts: Derivation Cohort and Validation Cohort [6]. The derivation cohort was used to develop a risk prediction tool from the 3.9% of patients who suffered a perioperative respiratory event of laryngospasm, bronchospasm (intra or postoperative) or postoperative apnea, hypopnea or prolonged oxygen requirement [6].

Age (≤3 years), ASA > 1, morbid obesity, preexisting pulmonary disorder, and anesthesia for surgery (versus radiology) were predictors of increased risk [6]. The Validation Cohort was then used to demonstrate the predictive validity of the tool. The discriminant performance of the final risk tool was most valuable in predicting those who would not have a high risk of a perioperative event. The tool had a negative predictive value of 98.2, positive predictive value of 5.8, sensitivity of 77.6 and specificity of 49.2 [6].

#### 5.1.3. The COLDS Score

The COLDS Score is a preoperative screening tool that has been validated in children < 6 years of age and intended to quantify the risk of PRCs. COLDS is an acronym for the identified risk factors for PRCs: C for current signs and symptoms; O for onset of symptoms; L for lung disease; D for the device to be used for airway management; and S for surgery type (whether it involves the airway or not, then further subclassified to major and minor airway) [22]. The COLDS score assigns each risk factor a numerical score of 1, 2 or 5 to quantify the risk as nil, mild or moderate/severe, respectively. A higher COLDS score would suggest more perioperative risk. The COLDs score has a high interrater reliability, most predictive in neonates and infants (age 0 to < 2 years) [22].

The COLDS score is most valuable in predicting bronchospasm, desaturation, the need for beta-agonist therapy and prolonged cough and less able to predict the occurrence of laryngospasm. The COLDS Score has been shown to be predictive of the cancellation of a planned surgical procedure for anesthesia-related concerns. In one study, all patients who had a COLD score of 19 and above were almost always cancelled [22].

### 5.2. Risk Factors for Perioperative Respiratory Complications

Table 2 summarizes the risk factors that have been identified with pediatric perioperative respiratory complications since 2000. The table has been structured to be used as a preoperative tool not only to identify risk factors (patient and procedure) but also possible modifications (modifiable and non-modifiable) to patient care which could be made to optimize outcome and minimize risk.

#### 5.2.1. Non-Modifiable Risk Factors

##### Age

**Figure 1:**
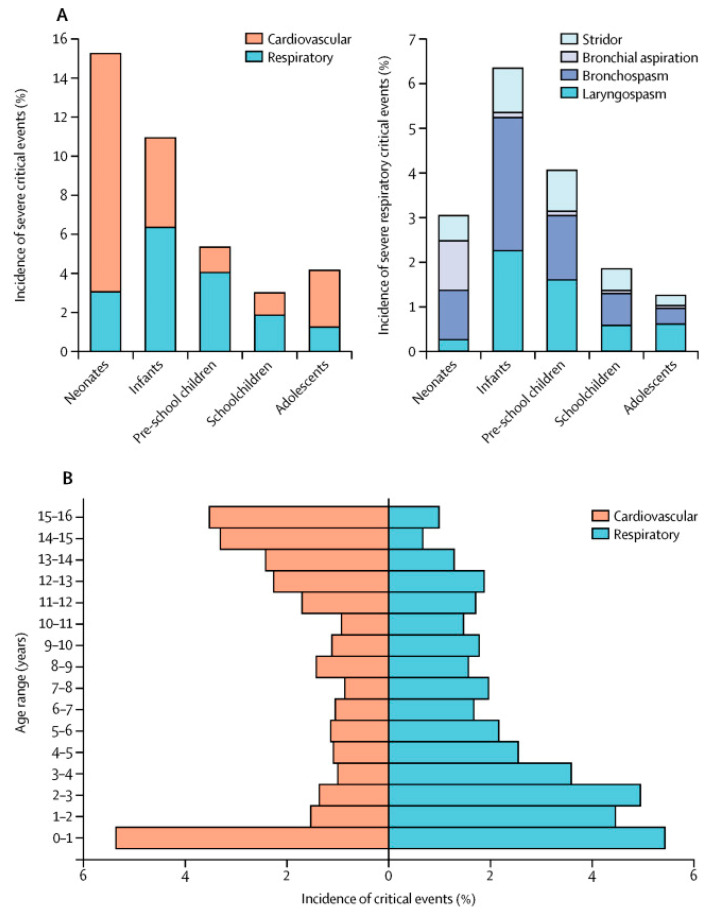
Distribution of severe critical events throughout the age groups. (**A**) Relative incidence and of respiratory and cardiovascular events (%) and the relative distribution of the four respiratory critical events (%). (**B**) Age distribution of cardiovascular (orange) and respiratory (blue) critical events [10].

Multiple pediatric studies have shown that the risk of PRCs decreases as the child ages [6,11,14,16,17,19]. Figure 1 shows the distribution of severe critical (respiratory and cardiovascular) events across pediatric age groups (0–16 years) in a prospective multicenter observational study in 261 hospitals in Europe [10]. Infants, particularly neonates, have the greatest risk of critical events intraoperatively and they tend to suffer primarily from PRCs [11,12,16,18,21,73,130,131]. Young and premature infants are at an even higher risk of PRCs as compared to full-term and older premature infants [130]. Studies have recommended that infants be at least 44 weeks post conceptual age for non-essential surgeries to decrease their risk of PRCs [132,133]. Kurth et al. expressed concern that preterm infants less than 60 weeks post-conceptual age were also at increased risk of PRCs [134]. These findings are in opposition with adult studies which, even after adjusting for co-morbidity, identify advancing age (age > 60 to 65 years) as a risk factor [7].

##### ASA Status

An American Society of Anesthesiologists Physical Status ≥ 3 increases the risk of PRCs by 4–14 fold in children [7,10,12,17,19,130,135].

Although the interrater reliability has not been as predictive as in the adult population, the ASA’s modification in 2014 of the ASA classification with approved clinical scenarios tailored to each definition, could improve the interrate reliability [136,137,138].

Despite the limitations of the ASA-PS classification system, an ASA score ≥3, alone or in combination with other clinical variables (co-morbidities, type of surgery, etc.) can guide the perioperative anesthetic care of the pediatric patient [6,10].

Although the overall incidence of medical malpractice claims in pediatrics is less than adults, the morbidity and mortality tend to be more significant: ASA 3-5 patients comprises only 23% of pediatric anesthesia closed malpractice claims. A total of 33% of these claims, however, resulted in death or brain damage, most commonly from respiratory and cardiovascular events [73,139].

##### Type of Surgery

Pediatric patients have been shown to be at greater risk of developing PRCs after otolaryngology (ENT) surgical procedures [14,16,17]. One study cites a 1.6 fold increase risk of PRCs after ENT surgery, with an odds ratio of 1.43 versus 2.74 for non-ENT and ENT procedures, respectively [16]. A total of two or more surgical procedures from different specialties has also been shown to increase the risk of critical events (the majority representing respiratory) in children by over 2-fold [11]. Pediatric procedures performed outside of the operating room (OOR) setting have been shown to pose up to a 2.71 increase risk, with a critical event rate of 1:814 versus 1:213 in OOR and operating room settings, respectively [6,11]. Further studies are required to determine whether patient demographics (ASA score, medical risk factors, age) or the setting (proximity to emergency aid, ancillary back-up services, equipment availability, familiarity of anesthesia staff with the OOR environment etc.) play a role in this significant discrepancy in risk.

Adult studies report differently, with abdominal and vascular surgeries representing higher risk for PRCs [7].

Both adult and pediatric literature report an increased risk of PRCs in emergent procedures. Although the degree of increased risk has not been estimated in children, the adult literature report a two- to six-fold increase in PRC risk as compared to elective procedures [7,12,18,135].

##### Preoperative Studies

Preoperative Labs, Chest X ray, Pulmonary Function Tests, Oxygen Saturation and Arterial Blood Gas

Preoperative evaluation of the pediatric patient should ultimately focus on minimizing the risk of PRCs by identifying any potential risk factors. Particularly, as we know that respiratory events (both upper and lower) account for a majority of pediatric PRCs, it is important to perform a thorough pulmonary and airway exam and review of systems. A routine preoperative laboratory assessment should be reserved for those whose medical or family history, review of systems or impending procedure, warrants focused preoperative testing [57,58,59].

There is no demonstrated value in obtaining routine chest X-rays in otherwise healthy pediatric patients who do not have baseline or acute lung disease, are not scheduled for major thoracic or abdominal surgery, do not smoke, are not on immunosuppressive therapy and do not have any risk factors for tuberculosis [67,68,69,70].

Similarly, pre-operative pulmonary function tests (PFTs) for those children who are scheduled for scoliosis and posterior spinal fusions, procedures with a presumptive high risk of perioperative complications and post-operative ventilation, have not been shown to be of value. A total of 30% of all children scheduled for these procedures were unable to deliver effective PFTs, and of the approximately 10% (37 of 357) who were found to be at high risk, only one remained intubated post-operatively. PFTs have not to-date been shown to be effective as a predictor for PRCs, post-operative intensive care unit admission or need for post-operative ventilation [71]. Similarly, spirometry has not been shown to be an effective predictor: a systematic review of blinded studies reported that 80% of spirometry values did not predict PRCs [72].

The role of preoperative oxygen saturation and arterial blood gas (ABG) tests has yet to be determined for the pediatric patient. Both have been shown to be helpful in predicting the risk of developing PRCs in adults [60,65,66]. Future studies are required to determine whether there is a predictive value for pre-operative oxygen saturations in children and, the possible role of ABG testing balanced with the logistical challenges which would be involved in obtaining them.

##### The Pediatric Difficult Airway

Difficult intubation, whether anticipated or unanticipated, has been shown to increase the risk of PRCs and perioperative cardiac arrest [10,15,74]. The Pediatric Difficult Intubation Registry (PeDI) reports that 20% of children with difficult tracheal intubations suffered at least one complication. PRCs comprised most non-severe complications and included transient hypoxemia (the most common), minor airway trauma, laryngospasm, and bronchospasm. Cardiac arrest occurred in 2% of the patients [74].

The PeDI registry was evaluated to find predictors of difficult intubations which was defined as requiring more than two attempts at direct laryngoscopy (DL): weight <10 kg, short thyromental distance, and three direct laryngoscopy (DL) attempts prior to deploying indirect technique. A difficult intubation was associated with high rates of failed intubation and up to a 30% incidence of severe complications. Strategies to minimize the risk of hypoxia and repeated intubations included pre-oxygenation via facemask and passive oxygen administration (nasal cannula) during active instrumentation of the airway. Early recognition of a difficult airway with rapid transition from direct to indirect laryngoscopy has been shown to be effective in a difficult airway situation [74].

#### 5.2.2. Modifiable Risk Factors

It is important to carefully evaluate the patient as well as the procedure to determine whether there are any opportunities to minimize the risk of PRCs either pre- or intra-operatively.

##### Patient Factors

Co-morbidity

Children with significant comorbidities and an ASA classification ≥ 3 have a higher likelihood of critical events in the perioperative period [6,10,151]. The American College of Surgeons introduced a National Surgical Quality Improvement Program (ACS NSQIP) as an initiative to minimize the surgical risks for children. They reported that comorbidities of nutritional/immune history (preoperative weight loss or chronic steroid use), physiologic compromise (sepsis, inotrope use prior to surgery) and ASA > 3 were associated with a higher likelihood of postoperative complications [151].

Preexisting pulmonary (odds ratio 3.95) and neurologic disease (OR 1.41) were also associated with increased PRC risk [6]. Sleep disordered breathing (SDB) has been noted to be associated with up to a two fold increase in PRC [10,22,152]. Airway insensitivity (history of wheezing, asthma diagnosis) has been reported to pose up to a threefold increased risk [10].

Congenital heart disease (CHD), even when undergoing non-cardiac surgery, is a risk factor for both cardiovascular (11.5%) and respiratory (4.7%) PRC in children. Non-cardiac surgery included otolaryngology, maxillofacial, gastroenterology and general surgical procedures. The more severe the CHD, the higher the risks of reintubation and mortality [30].

Children with asthma should be optimized from a respiratory standpoint prior to surgery. All asthma medications should be continued up to and including day of surgery and corticosteroids (inhaled or systemic) and short-acting inhaled beta agonists pre-operatively [153,154,155].

Obstructive sleep apnea poses an inherent risk for PRCs in children. A detailed assessment of diagnostic sleep studies, home monitors (pulse oximeter, apnea monitor) as well as non-surgical home medical management (supplemental oxygen, bi-level positive airway pressure [BiPAP], continuous positive airway pressure [CPAP], preferred sleep position) should be obtained [156]. These details will aid in the planning of pre, peri and postoperative management. Home CPAP and BiPAP machines should accompany the child on day of surgery, and in some cases, may be used in the post-operative and recovery period as well as in the transport home after discharge [156].

Upper Respiratory Infection (URI)

There is a significantly increased risk of PRCs in children with acute or recent (<2 weeks) upper respiratory infection (URI) [14,22,27,28,29]. The precise time of risk following a URI remains unclear: Some studies suggest that airway inflammation and hypersensitivity can last up to several weeks, while others advocate that the risk is for only the first two weeks after the beginning of URI symptoms. Tait et al. found a higher risk of PRCs in patients with active or recent URI to last up to 4 weeks after URI symptoms resolve; however, none of the PRCs led to any long-term adverse sequelae [29]. The consensus of most pediatric anesthesiologists is that elective surgery be delayed until 2–3 weeks following resolution of URI symptoms [27,28].

In the event that an anesthetic is necessary for a child with an active URI, there are increased risks of PRC with endotracheal intubation, age < 5 years, history of prematurity, history of reactive airway disease, parental smoking, surgery involving the airway, presence of copious secretions, and nasal congestion. An endotracheal intubation increased the likelihood of breathholding, severe cough, and desaturation < 90%. When comparing ETT to LMA in these URI patients, the ETT was associated with a higher rate of significant desaturation but had similar rates of cough and breathholding as LMA [29].

Obesity

Up to one-third of pediatric surgical patients suffer from obesity [11,44,45,46]. Childhood obesity is linked to many other major comorbidities, similar to adults, like: hypertension, type II diabetes, heart disease, hyperlipidemia, and asthma [47,48,49,50]. Obesity increases the likelihood of apnea/hypopnea, a difficult mask airway, laryngospasm (the most common PRC in children), severe desaturation, cardiac arrest and an increased postoperative oxygen requirement [6,52,53,54,55]. Sleep disordered breathing is more common with pediatric obesity ranging from snoring to obstructive sleep apnea (OSA) [51]. The APRICOT study, a prospective observational multicenter European cohort identified the incidence, nature, and outcome of severe critical events in children in the perioperative period. Snoring increased the risk of severe critical events (most of which were PRCs) by two-fold [10].

In adults, weight loss of 5%–10% has been shown to significantly reduce the perioperative risk of complications from chronic comorbidities often associated with obesity. Although no such study has been done in children, consideration should be given to weight reduction pre-operatively, especially in morbidly obese children presenting for procedures who also have existing risk factors predisposing them to PRC [56].

Smoking/Passive SMOKING/vaping

Passive smoking (exposure to cigarette smoke) is associated with increased risk (relative risk RR 1.39) of PRCs [10,29]. Children of smokers have a higher incidence of breathing problems, airway reactivity, asthma exacerbation and PRCs, particularly in children with active URI (RR 1.6) [29,31,32,33,34,35].

In adults, a meta-analysis comparing current smokers and ex-smokers of at least 4 weeks or greater showed a significant reduction in PRCs for ex-smokers (RR 0.81) [36]. Smoking cessation for >4 weeks and >8 weeks pre operatively can decrease the incidence of PRCs by 23% and by 47%, respectively [36,37]. An active smoking intervention program demonstrates a higher cessation rate (up to 25%) compared to those without active engagement (8%) [38]. With these adult studies in mind, active counseling and intervention should be attempted in adolescents and children who smoke, as well as of their parents who are exposing them to passive smoke [39].

Electronic cigarettes (e-cigarettes)/vaping devices (both nicotine and, less commonly, non-nicotine) have not been shown to have benefits in either short- or long-term cessation [40]. The prevalence of vaping amongst adolescents in 8th, 10th, and 12th grade has more than doubled between 2017 and 2019 (3.5 to 9, 8.2 to 20.2, and 11.0 to 25.4, respectively) [41]. This is particularly concerning given the potential link recently described between vaping and acute lung injury [42]. Airway burns created by the inhaled chemical agents can lead to a constellation of pathophysiologic signs similar to Acute Respiratory Distress Syndrome (ARDS): inflammation, airway edema with sloughing of the epithelium, alveolar inflammation, and hypoxemia [43]. Preoperative screening for vaping/e-cigarette use and its potentially associated symptoms amongst early teens/adolescents is warranted.

Preoperative Anemia and Blood Transfusion

Anemia is reported in up to 17% of children presenting for routine preoperative screening for elective surgery [61]. Although a higher incidence of in-hospital mortality has been reported in anemic neonates and children presenting for non-cardiac surgery, it is important to consider this in the context which was extremely ill children for complex surgical procedures [62,63]. Preoperative anemia, defined as hematocrit <40% for neonates and <30% for infants >30 days old as well as toddlers to adolescents, in otherwise healthy children < 1 year of age has not been associated with PRCs in the setting of uncomplicated surgeries [64].

The value of preoperative transfusions in the setting of anemia remains unclear. In adults, this practice has not been shown to decrease the risk of developing PRCs in this at-risk population. Rather, transfusions have increased the risk of PRC [60,76,93,94]. A systematic review with meta-analyses of randomized controlled trials comparing a restrictive versus liberal transfusion strategy in both adult and pediatric patients reported no differences in mortality or morbidity between the two strategies, despite reduced number of packed red blood cells used and less patients transfused [94]. The general consensus is that the underlying cause of the anemia should be determined and treated if possible in order to minimize the risk of transfusion-related PRC [64,95,96].

##### Procedure Factors

General Anesthesia versus Sedation

In adults, general anesthesia (GA) has been shown to be an independent risk factor for PRCs, a risk which is decreased with regional anesthesia [60,76,77].

The APRICOT study demonstrated a 3-fold increase in the risk of severe respiratory critical events (PRCs) with GA versus sedation (Relative Risk 3.15) [10]. In children, the risk of PRCs has been shown to be higher with midazolam premedication, desflurane (versus sevoflurane), and in those whose vocal cords were sprayed with lignocaine [14]. Inhalation anesthesia increases the risk of laryngospasm more than monitored anesthesia care with propofol [14]. Endotracheal intubation increases the risk of coughing and bronchospasm and poses a higher risk of PRCs as compared to anesthesia delivered by face mask, particularly in the asthmatic and recent URI at-risk population [14,18,88]. von Ungern-Sternberg et al., found that the insertion of a cuffed versus uncuffed endotracheal tube should be carefully considered in children. The uncuffed endotracheal tube increases the risk of laryngospasm (10 vs 3%) and stridor (4% versus 0%) [14].

There has been no reported difference in extubation techniques relative to PRCs. Deep versus awake extubations have similar rates of PRC, despite the risk of coughing with concomitant oxygen desaturation, postoperative stridor and sore throat which are risks with awake extubations [157,158]. In general, children with difficult airway, obesity and aspiration risk should be extubated awake while consideration should be given to deep extubations in those with increased airway sensitivity (asthma, URI) [18,159].

Long-Acting Neuromuscular Blocking Drugs and Sugammadex

The role of neuromuscular blocking drugs (NMBD) and reversal has not been extensively studied in children. In adults, neostigmine has been shown to be an independent risk factor for PRC, the exact etiology of which is still unclear [7,81].

Sugammadex is a more recent neuromuscular reversal agent that specifically targets aminosteroidal NMBDs [82,83]. A systematic review of randomized controlled trials reported that sugammadex produces more rapid neuromuscular reversal with less bradycardia, as compared to neostigmine and placebo [84]. The use of a nerve stimulator is important to verify the reversal of neuromuscular blockade and has been shown to be an independent factor in decreasing the risk of PRC [81,85,86,87].

Anesthesiologist: Pediatric Subspecialty-Trained versus General/ Experienced versus Junior

There is a general consensus in the literature that pediatric subspecialty training reduces the rate of PRCs [10,16,19,75].

A study comparing the incidence of adverse respiratory events under the care of pediatric and non-pediatric trained anesthesiologists, reported that 80% of laryngospasm (23 out of 29 cases) and 80% of airway obstruction (47 out of 58 cases) occurred when a specialized pediatric anesthesiologist was not present. PRCs were approximately twice as frequent when the anesthesia provider was an anesthesia physician trainee [16]. The PeDI airway registry reports that in cases with five endotracheal intubation attempts, pediatric anesthesiologists were responsible for 44% of the successful attempts despite making the first airway attempt in 21%. The recommendations were that after a failed first attempt at intubation by a trainee or less experienced airway provider, there should be rapid consideration given to switching providers and giving the airway to the most experienced [74].

A large review of institutional audits, closed claims, and large-scale studies on pediatric perioperative cardiac arrest, suggested that for children < 3 years age, the risks of minor and major morbidities decrease in those with significant experience caring for this age group. Those with additional training or focused expertise in pediatric airway management, vascular access and management of massive hemorrhage are less likely to suffer pediatric perioperative cardiac arrests as compared to their less experienced peers [73].

Open versus Laparoscopic

In adults, laparoscopic surgery tends to reduce postoperative respiratory dysfunction. For laparoscopic cholecystectomy (LC), postoperative forced vital capacity (FVC) dropped to 73% of preoperative versus 52% for open cholecystectomy (OC). Forced expiratory volume (FEV1) dropped to 53% and 72% of baseline preoperative function for OC versus LC, respectively [89].

A meta-analysis of randomized clinical trials demonstrated that laparoscopic inguinal hernia repair, despite the longer surgical time, had earlier hospital discharge, faster return to baseline function and work, and significantly fewer postoperative complications than open repair [90]. More recent laparoscopic studies support these findings. Laparoscopic splenectomies, despite longer operating times, are associated with reduced morbidity and fewer PRCs [91].

To date, there have been no comparative studies done in pediatrics, for difference in PRC. The National Surgical Quality Improvement Project reported that in patients < 21 years, laparoscopic proctocolectomy despite having a longer operating time and increased risk of surgical infection, had shorter postoperative hospital length of stay and reduced risk of superficial surgical site infections (SSI) [92].

The decision and choice to perform open versus laparoscopic procedures should be carefully weighed, considering patient acuity, skill of surgeon, pre-existing pulmonary status and balancing these factors with the increased risk of PRCs with longer surgical times. Longer surgical time is a known risk [10,11,78,79,80].

Mechanical Ventilation Strategy

Intraoperative ventilation strategy designed to protect pulmonary function is critical to minimizing patient risk. Although the recommendations were drawn from adults, they are logically applicable to pediatric patients. Moderate to high positive end-expiratory pressure [PEEP], low tidal volume, peak inspiratory pressure [PIP] <30 cmH2O, and avoidance of high FiO2 are all effective strategies, adopted from ARDS experience, in the prevention of PRCs in perioperative medicine [97,98,99,100,101,102].

Moderate to High Peep

While the use of moderate to high PEEP is broadly accepted and common practice in pediatric ICUs, this may not translate to the operating room environment as adult studies suggest that low tidal volumes with moderate PEEP (6–8 cm H2O), may reduce PRCs. There is actually some concern that high PEEP may be harmful in adults [103,104]. The PROVHILO study was a randomized control trial (RCT) that compared high PEEP (12 cm H2O) combined with low tidal volume and recruitment maneuver (RM) versus low tidal volume, low PEEP (≤2 cm H2O) without RM. The high PEEP group suffered hemodynamic consequences and a 40% rate of PRCs. The low PEEP group did not manifest hemodynamic compromise and had a similar PRC rate of 39% [104]. Fluid resuscitation prior to high PEEP and RM has been shown to mitigate hemodynamic compromise associated with high PEEP [105]. A retrospective study of 64,000 ventilated adult non-cardiac surgical patients demonstrated that PEEP of 5 cm H2O combined with plateau pressure of ≤16 cm H2O, had fewer PRCs than zero PEEP [101].

Future studies are needed, specifically for the pediatric population, to determine the relationship between tidal volume, PEEP and PRC [100,102,106].

While the optimal PEEP is not exactly known, the current available adult studies do support the use of some level of moderate PEEP for the prevention of atelectasis and should be considered for pediatric patients without lung disease, reserving high PEEP for those with pulmonary pathology [24,107,108,109,110,111,112,113,114].

Low Tidal Volume

The data on low tidal volume in pediatric surgical patients is sparse and does not provide definitive guidance. A meta-analysis of observational studies in mechanically ventilated children reported no relationship between tidal volume and clinical outcomes [115]. Most recommendations for tidal volume strategy in pediatrics are based on adult data which suggest tidal volume be kept <10 ml/kg [115,116,117,118,119,120,121].

Peak Inspiratory Pressure (PIP) < 30 cm H2O

The maintenance of PIP < 30 cm H2O is now common practice in pediatric surgical patients, like in adults, as adopted from ARDS network recommendations [99,122].

Avoidance of High FiO2

In general, the intraoperative FiO2 is kept to the minimum required for maintenance of sufficient oxygenation (oxygen saturation of 92% and above) [123,124,125]. Critical care literature has demonstrated worsened lung function with excessive oxygen supplementation in mechanically ventilated patients with acute lung injury [126]. In neonatal medicine, the oxidative stress of hyperoxia is responsible for significant end organ injuries to the lungs (bronchopulmonary dysplasia), eyes and brain [25,127,128,129]. Although the mechanism of pulmonary injury in the neonates is not clearly established, in the geriatric population, hyperoxia triggers pro-inflammatory cytokines that lead to permanent destruction of elastic fibers of the lungs [25]. A current recommendation is that oxygen delivery be tailored to the patient and procedure. Although FiO2 80% may be used for induction and emergence phases, a FiO2 of 25–35% for infants and children, 60–80% for middle-aged, and 30–40% for elderly patients is recommended for the maintenance phase [25].

## 6. Postoperative Respiratory Concerns

Tools to assess the risk of postoperative respiratory failure.

The prediction of postoperative respiratory failure is an important focus of study as it has significant impact on healthcare costs, morbidity and mortality [1,2,4,160,161]. Four risk prediction tools have been developed to stratify the risk of postoperative respiratory failure. Although only one of the tools has been validated in children, understanding the other three tools is important and may have applications in pediatric care.

### 6.1. Prediction Tool to Determine the Need for and the Duration of Use of Postoperative Oxygen Therapy

This is the only pediatric prediction tool. It was developed from a case-control study of 9820 children (<15 years of age) who underwent GA between 2010 and 2013 in a tertiary hospital in Thailand. The primary outcomes were the need for and duration of postoperative oxygen therapy. Risk scores were classified into high (score ≥ 12), intermediate (8–11), or low (≤7) risk groups. The tool demonstrated high predictive ability. The common risk factors for oxygen requirement postoperatively were probable difficult airway, ASA physical status ≥ 3, and surgeries involving the airway, thorax, and abdomen [162].

### 6.2. The Assess Respiratory Risk in Surgical Patients in Catalonia Score (ARISCAT)

The ARSICAT score assesses seven easily obtainable clinical factors: age (adult patients age 55 to 80 years), preoperative oxygen saturation in room air, respiratory infection in the last month, preoperative anemia, upper abdominal or intrathoracic surgical incision, duration of surgery, and emergency procedures. This scoring tool is validated to be able to stratify risk of PRCs into three levels—low risk (<26 points), moderate risk (26–44 points), and high risk (>45 points) [60]. The definitions of PRCs used for the ARISCAT study were developed from the 2015 European joint task force guidelines for perioperative outcome (EPCO): respiratory failure, suspected pulmonary infection, pleural effusion, atelectasis, pneumothorax, bronchospasm and aspiration pneumonia [9]. This tool has not been validated in children.

### 6.3. GUPTA Risk Calculator Predicting Postoperative Respiratory Failure

The American College of Surgeons National Surgical Quality Improvement Program (NSQIP) reviewed a multicenter prospective dataset from 2007 to 2008 to elicit the preoperative factors that correlate with increased risk of postoperative respiratory failure. Gupta et al. then used these factors to develop and validate a risk calculator to predict postoperative respiratory failure. The preoperative factors found to be associated with increased risk of postoperative respiratory failure were type of surgery, emergency case, dependent with functional status, sepsis, and higher ASA class [163]. This risk calculator has not been validated in children.

### 6.4. Score for Prediction of Postoperative Respiratory Complications (SPORC)

The SPORC tool was developed and validated from electronic records and billing information of adults with the goal of predicting the risk of postoperative reintubation (within the immediate 3 postoperative days) with a subsequent need for mechanical ventilator support. An 11-point score was developed from independent predictors. The factors that were found to predict postoperative reintubation were: ASA score of ≥3, emergency surgery, high-risk surgical service, history of congestive heart failure, and chronic pulmonary disease [164].

### 6.5. Postoperative Pain Management

The first 24 h of the postoperative phase is the period with the highest likelihood of respiratory failure associated with the administration of Analgesics—specifically opioids [165,166,167]. Studies have demonstrated that neuraxial analgesia, when combined with GA, reduces the risk of PRCs (hypoventilation, postoperative ventilation, postoperative reintubation and postoperative pneumonia) when compared to opioids alone, especially in patients with underlying respiratory pathology [92,165,166,167,168,169].

Patients with obesity-related OSA are known to have higher rates of postoperative hypoventilation and oxygen desaturation due to hypersensitivity to opioids; therefore, opioid-sparing techniques are highly recommended for postoperative pain management in patients with obesity [170,171,172].

Adequate pain control is necessary to prevent splinting and the development of atelectasis and desaturation [173].

### 6.6. Physical Therapy: EARLY Mobilization and Chest Physiotherapy

Early mobilization has been shown to be feasible and safe in critically ill children, however, the efficacy of mobilization in shortening duration of mechanical ventilation and ICU duration of stay remains unclear [174,175].

Chest physiotherapy has not been shown to reduce the rate of PRCs [173]. The I COUGH initiative (incentive spirometry, coughing and deep breathing, oral care, understanding, getting out of bed, and head of bed elevation) has been widely instituted in ICUs. Preliminary data from this initiative suggests that the rate of pneumonia and unplanned reintubation in postoperative adults is lowered [173,176]. The role of combining adequate pain management, physical therapy, early mobilization and good oral care in order to minimize postoperative PRCs will require further large scale studies [173].

The applicability of this I COUGH initiative to the pediatric population warrants future study.

## 7. Conclusions

Perioperative respiratory complications in pediatric patients are quite common and lead to increase in hospital length of stay and health care costs. While there are perioperative risk prediction tools that measure PRC risk, few are pediatric-specific, and they can be quite difficult to apply in clinical practice. Routine preoperative laboratory assessment should be reserved for those whose medical or family history, review of systems or impending procedure, warrants focused preoperative testing. The role of preoperative oxygen saturation and ABG has yet to be determined for the pediatric patient, though both have been shown to be helpful in predicting the risk of developing PRCs in adults. Early recognition of a difficult airway with rapid transition from direct to indirect laryngoscopy has been shown to be effective in a pediatric difficult airway situation. Children with significant comorbidities and an ASA classification ≥ 3 have a higher likelihood of critical events in the perioperative period. Children with asthma should be optimized from a respiratory standpoint prior to surgery. Obstructive sleep apnea also poses an inherent risk for PRCs in children. A detailed assessment of diagnostic sleep studies, home monitors (pulse oximeter, apnea monitor) as well as non-surgical home medical management (supplemental oxygen, CPAP, BiPAP, preferred sleep position) should be obtained and should be made available for in-hospital use during the immediate postoperative period. There is a significantly increased risk of PRCs in children with acute or recent (<2 weeks) URI. The precise time of risk following a URI remains unclear though the consensus amongst pediatric anesthesiologists is that elective surgery be delayed until 2–3 weeks following resolution of URI symptoms. Childhood obesity is linked to many major comorbidities such as hypertension, type II diabetes, heart disease, hyperlipidemia, and asthma and it increases the likelihood of PRCs in children, like adults. Both passive and active smoking increase the risk of PRCs in children and preoperative screening for vaping/e-cigarette use and its potentially associated symptoms amongst early teens/adolescents is necessary given the increased prevalence of vaping in that age group. The underlying cause of anemia should be determined and treated if possible, to minimize the risk of transfusion-related PRC. It is important to note that that pediatric subspecialty training reduces the rate of PRCs. Intraoperative ventilation strategy designed to protect pulmonary function is critical to minimizing patient risk. Moderate to high PEEP, low tidal volume, PIP < 30 cm H2O, and avoidance of high FiO2 are all effective strategies, adopted from ARDS experience, in the prevention of PRCs in perioperative medicine. Neuraxial analgesia, when combined with GA, reduces the risk of PRCs when compared to opioids alone especially in patients with underlying respiratory pathology and obesity-related OSA. Vigilance to the various factors, both modifiable and non-modifiable, that are associated with the increased risk of PRCs should support detailed preoperative assessment, more accurate informed consent, optimized intraoperative management strategy and improved allocation of clinical resources in order to decrease morbidity and mortality.

## Figures and Tables

**Table 1:** Incidence of perioperative respiratory complications in major pediatric studies since the year 2000.

STUDY	YEAR	DESIGN	PRC(s) Described	Sample Size	PRC ^c^ Incidence n (%)	Surgical Specialty
Habre et al. [10]. (APRICOT ^a^ Study)	2017	Prospective, Multi-Center Cohort	PRCs ^c^ **Laryngospasm** **Bronchospasm** **Bronchial Aspiration** **Stridor**	31127	Severe Critical Events 1637 (5.3) PRCs ^c^ 976 (3.1) **Laryngospasm** 368 (1.2) **Bronchospasm** 371 (1.2) **Bronchial Aspiration** 29 (0.9) **Stridor** 208 (0.6) *Incidence of Cardiac Instability 597 (1.9) **Most of the Severe Critical Events were PRCs ^c^ 59.6%	Multi-Specialty, Elective Surgery Only, Ambulatory Surgery Only
Schleelein et al. [11]	2016	Retrospective, Case-Control	PRCs ^c^ **Laryngospasm** **Bronchospasm** **Difficult Ventilation** **Airway Obstruction** **Desaturation** **Respiratory failure after extubation** **Breath holding** **Other**	55070	Anesthesia Now! 213 (0.4) PRCs ^c^: 143 (0.26) **Laryngospasm** 63 (0.11) **Bronchospasm** 20 (0.04) **Difficult Ventilation** 18 (0.03) **Airway Obstruction** 12 (0.02) **Desaturation** 11 (0.02) **Respiratory failure after** **extubation** 11 (0.02) **Breath holding** 7 (0.01) **Other** 5 (0.01) *Most of the Anesthesia Now! Events (67%) were Respiratory Events (PRCs ^c^) **10.8% of the Anesthesia Now! Events were Cardiac Events.	Multi-specialty, Elective and Emergency
Subramanyam et al. [6]	2016	Prospectively Collected Data, Retrospective Cohort Analysis, Single Center	PRCs ^c^ Intraoperative **Laryngospasm** (Requirement of PPV > 20cm H2O or Administration of Succinylcholine) **Bronchospasm** (Use of Albuterol) Postoperative **Laryngospasm** (Requirement of PPV > 20cm H2O or Administration of Succinylcholine) **Bronchospasm** (Use of Albuterol) **Apnea/Hypopnea** (Need of Bag Mask Ventilation) **Oxygen Requirement** (Oxygen Need 2 h Post op to Keep Sats > 92%)	19059	520 (2.8) Intraoperative **Laryngospasm** 119 (0.7) **Bronchospasm** 68 (0.4) Postoperative **Laryngospasm** 74 (0.4) **Bronchospasm** 36 (0.2) **Apnea/Hypopnea** 105 (0.6) **Oxygen Requirement** 255 (1.5)	Multi-Specialty, Elective Surgery Only, Ambulatory Surgery Only
de Graaff et al. [12]	2014	Retrospective Single-Center Cohort	4 out of 20-Item Complication List were PRCs ^c^: **Aspiration** **Bronchospasm** **Laryngospasm** **Hypoxemia** **Hypoventilation**	35190	1195 Critical Incidents (3.4) 564 (46.5) Respiratory Complications **Aspiration**: 19 (0.05) **Bronchospasm**: 60 (0.2) **Laryngospasm**: 257 (0.7) **Hypoxemia**: 146 (0.4) **Hypoventilation**: 92 (0.3)	Multi-Specialty, Elective, and Emergency
Kurth et al. [13] (Wake-Up Safe)	2013	Prospective, Multi-Center Cohort	**Respiratory Events**	736365	Serious Adverse Events 740 (0.1) **Respiratory Events**: 254 (0.03) *Most of the SAE’s ^d^ (34%) were Respiratory Events (PRCs ^c^)	Multi-Specialty, Elective and Emergency
Von Ungern-Sternberg et al. [14]	2010	Prospective, Single-Center Cohort	PRCs ^c^ **Bronchospasm** **Laryngospasm** **Cough** **Desaturation < 95%** **Airway Obstruction**	9297	1392 (15) **Bronchospasm** 193 (2) **Laryngospasm** 351 (4) **Cough** 687 (7) **Desaturation** < 95% 919 (10) **Airway Obstruction** 332 (4)	Otolaryngology, Elective and Emergency
Bhananker et al. [15] (POCA ^b^ Study)	2007	Prospective, Multi-Center Cohort	Perioperative Cardiac Arrests between 1998–2004 PRCs ^c^ **Laryngospasm** **Other Airway Obstruction** **Inadequate ventilation/Oxygenation** **Inadvertent/premature extubation** **Difficult Intubation** **Esophageal or endobronchial intubation** **Bronchospasm** **Pneumothorax** **Aspiration** **Other** **Presumed respiratory, unclear mechanism**	193	PRCs ^c^ 53 (27) **Laryngospasm** 11 (6) **Other Airway Obstruction** 5 (3) **Inadequate ventilation**/**Oxygenation** 9 (5) **Inadvertent/premature extubation** 7 (4) **Difficult Intubation** 4 (1) **Esophageal or endobronchial Intubation** 3 (2) **Bronchospasm** 4 (2) **Pneumothorax** 2 (1) **Aspiration** 2 (1) **Other** 1 (1) **Presumed respiratory, unclear mechanism** 5 (3)	Multi-specialty, Elective and Emergency
Mamie et al. [16]	2004	Prospective, single Center Cohort	PRCs ^c^ **Laryngospasm** (Complete Airway Obstruction Associated with Muscle Rigidity of Abdominal Wall or Chest Wall, Unrelieved by Maneuvers to Relieve Soft Tissue Obstruction) **Bronchospasm** (Increase in Respiratory Effort, Especially Expiration, Associated with Hypercapnia and Oxygen Desaturation, Wheeze on Auscultation, Capnography Changes in Ventilated Patients with Increase in the Slope of the Plateau, and Increase in Airway Peak Pressure) **Airway Obstruction** (Partial Airway Obstruction with Snoring Noise and Respiratory Efforts Without Deep Desaturation; Relieved Easily by Jaw Thrust, Positive Airway Pressure and/or a Guedel Airway) **Oxygen Desaturation** (SpO2 < 95%) **Recurrent Cough**	757	PRCs ^c^ 211 (27.9) **Laryngospasm** 30 (4.2) **Bronchospasm** 12 (1.6) **Airway obstruction** 69 (9.3) **Oxygen Desaturation** 69 (9.3) **Recurrent Cough** 146 (19.8)	Multi-Specialty, Elective Surgery Only
Murat et al. [17]	2004	Prospective, Single Center Cohort	**PRCs** ^c^ **Bronchospasm** **Hypercarbia** **Hypoxemia** **Aspiration** **Laryngospasm** **Reintubation**	23043	*Most of the intraoperative adverse events (53%) were respiratory events (PRCs^c^) **Intraoperative** **Adverse Events** 724 (3.1) 1105 (4.8) **PRCs**^c^ 383 (1.7) 242 (1.1) **Bronchospasm** 48 (0.2) 20 (0.1) **Hypercarbia** 19 (0.1) 18 (0.1) **Hypoxemia** 170 (0.7) 70 (0.3) **Aspiration** 10 (0.05) 9 (0.05) **Laryngospasm** 57 (0.25) 11 (0.05) **Reintubation** 37 (0.16) 24 (0.1)	Multi-specialty, Elective and Emergency surgery
Budic et al. [18]	2004	Prospective, Single Center Cohort	*Adverse Respiratory Events were Defined as any Episode of Perioperative Airway Obstruction (e.g., Laryngospasm), Oxygen Desaturation Less than 90% (for ≥ 10 s), Breath Holding (≥ 15 s), Severe Coughing, and Any Requirement for Unanticipated Endotracheal Intubation. PRCs ^c^ **Laryngeal Spasm** (Complete Obstruction) **Laryngeal Inspiratory Stridor** (Partial) **Irregular Breathing** (Breath Holding) **Apnea** **Cough** **Hiccup** **Excess Secretions** **Obstruction by Tongue**	682	PRCs ^c^ 39 (5.71) **Laryngeal Spasm** 3 (0.44) **Laryngeal Inspiratory Stridor** 15 (2.2) **Irregular Breathing** 10 (1.47) **Apnea** 3 (0.44) **Cough** 3 (0.44) **Hiccup** 3 (0.44) **Excess Secretions** 1 (0.14) **Obstruction by Tongue** 1 (0.14)	Multi-Specialty, Elective and Emergency Surgery
Tay et al. [19]	2001	Prospective, Single Center Cohort	PRCs ^c^ **Hypoxia** **Laryngospasm** **Bronchospasm** **Pulmonary Aspiration**	10000	297 Damaging Events Described *Most of the Damaging Events (77%) were Respiratory Events (PRCs ^c^) PRCs^c^ 230 (77.44) **Hypoxia** 101 (34) **Laryngospasm** 106 (35.7) **Bronchospasm** 20 (6.7) **Pulmonary Aspiration** 3 (1)	Multi-Specialty, Elective and Emergency Surgery

^a^ APRICOT, Anaesthesia Practice in Children Observational Trial; ^b^ POCA, pediatric perioperative cardiac arrest; ^c^ PRC, perioperative respiratory complication; ^d^ SAE, serious adverse event. **Bold font** denotes a PRC.

**Table 2:** Published risk factors for developing perioperative respiratory complications in pediatric patients categorized into patient factors and procedure factors which are both sub-categorized into non-modifiable and modifiable.

PATIENT FACTORS	PROCEDURE FACTORS
Modifiable	Modifiable
Lung Disease: Asthma [14,23] Pneumonia [24] Chronic Lung Disease [25,26] URI ^n^ [14,22,27,28,29] Congenital Heart Disease [30] Smoking/Passive Smoking/Vaping [10,29,31,32,33,34,35,36,37,38,39,40,41,42,43] Obesity [6,11,44,45,46,47,48,49,50,51,52,53,54,55,56] Laboratory/Clinical Test Findings [57,58,59] Preoperative Anemia [60,61,62,63,64] Preoperative Oxygen Saturation [60,65,66] Preoperative CXR ^c^ findings [67,68,69,70] Abnormal PFT’s ^k^ (FEV 1^d^/FVC ^e^ Ration < 0.7 and FEV1^d^ < 80% of predicted) [71,72]	Pediatric Anesthesiologist versus General [10,16,19,73,74,75] GA ^f^ Versus Regional [10,14,60,76,77] Duration of Procedure (Long versus Short ) [10,11,78,79,80] Re-Operation [11] Complexity of Procedure (Simple versus Complex) [11] Location (OR ^i^ versus Remote Location) [6,11] Long-Acting NMBDs ^h^ [7,81] Sugammadex [81,82,83,84,85,86,87] Supraglottic Airway Versus Endotracheal Airway [14,18,88] Open Versus Laparoscopic Abdominal Surgery [89,90,91,92] Intraoperative Blood Product Transfusion [76,93,94,95,96] Mechanical Ventilation Strategy [7,24,25,97,98,99,100,101,102,103,104,105,106,107,108,109,110,111,112,113,114,115,116,117,118,119,120,121,122,123,124,125,126,127,128,129]
**Non-Modifiable**	**Non-Modifiable**
Age (Preemies, Infants, Young Children) [6,11,14,16,17,18,19,21,73,130,131,132,133,134] ASA ^a^ III or higher [7,10,12,17,19,130,135,136,137,138,139] Difficult Airway (NEAR4Kids ^g^) [74] Head Injury/TBI ^l^ [140,141] Craniofacial Anomalies [142]	Type of Surgery OSA^j^/Adenotonsillectomy [14,16,17] Head and Neck [14,16,17] Pyloric Stenosis [64] TEF ^m^ [143,144] CDH ^b^ [145] Gastroschisis [146,147] Omphalocele [146,147] Neurosurgery [148,149] Posterior Spinal Fusion [71,150] Elective versus Emergency [7,12,18,135]

^a^ ASA, American Society of Anesthesiologists; ^b^ CDH, congenital diaphragmatic hernia; ^c^ CXR, chest x-ray; ^d^ FEV1, forced expiratory volume in 1 second; ^e^ FVC, forced vital capacity; ^f^ GA, general anesthesia; ^g^ NEAR4kids, National Emergency Airway Registry for Children; ^h^ NMBDs, neuromuscular blocking drugs; ^I^ OR, operating room; ^j^ OSA, obstructive sleep apnea ; ^k^ PFTs, pulmonary function tests; ^l^ TBI, traumatic brain injury; ^m^ TEF, tracheoesophageal fistula; ^n^ URI, upper respiratory illness.

## Abbreviations

**ABG**	arterial blood gases
**ACS NSQIP**	The American College of Surgeons National Surgical Quality Improvement Program
**AHRQ**	the Agency for Healthcare Research Quality
**APRICOT**	Anaesthesia Practice In Children Observational Trial
**ARDS**	acute respiratory distress syndrome
**ARISCAT**	Assess Respiratory Risk in Surgical Patients in Catalonia
**ASA**	American Society of Anesthesiologists
**ASA-PS**	American Society of Anesthesiologists Physical Status
**BiPAP**	bi-level positive airway pressure
**CDH**	congenital diaphragmatic hernia
**CHD**	congenital heart disease
**CPAP**	continuous positive airway pressure
**CXR**	chest x-ray
**DL**	direct laryngoscopy
**ENT**	Ear, Nose, and Throat
**EPCO**	European perioperative clinical outcomes
**ETT**	endotracheal tube
**FEV1**	forced expiratory volume in 1 second
**FiO2**	fraction of inspired oxygen
**FVC**	forced vital capacity
**GA**	general anesthesia
**I COUGH**	incentive spirometry, coughing and deep breathing, oral care, understanding, getting out of bed, and head of bed elevation
**ICU**	intensive care unit
**LC**	laparoscopic cholecystectomy
**LMA**	laryngeal mask airway
**NEAR4kids**	National Emergency Airway Registry for Children
**NMBDs**	neuromuscular blocking drugs
**OC**	open cholecystectomy
**OOR**	outside of the operating room
**OR**	odds ratio
**OR**	operating room
**OSA**	obstructive sleep apnea
**PeDI**	pediatric difficult airway
**PEEP**	positive end expiratory pressure
**PFTs**	pulmonary function tests
**PIP**	peak inspiratory pressure
**POCA**	pediatric perioperative cardiac arrest
**PRAE**	perioperative respiratory adverse event
**PRC**	perioperative respiratory complication
**PROVHILO**	protective ventilation using high versus low positive end-expiratory pressure
**PSG**	polysomnography
**PSI**	patient safety indicators
**RM**	recruitment maneuver
**SAE**	serious adverse event
**SDB**	sleep disordered breathing
**SPORC**	Score for Prediction of Postoperative Respiratory Complications
**SRBD**	sleep-related breathing disorder
**SSI**	surgical site infection
**STUBR**	snoring, trouble breathing, and un-refreshed
**TBI**	traumatic brain injury
**TEF**	tracheoesophageal fistula
**URI**	upper respiratory illness
**WUS**	wake-up safe
